# Microbiological Study of Cast Posts before Cementation

**DOI:** 10.1155/2017/1090534

**Published:** 2017-02-20

**Authors:** Maricela Vallejo-Labrada, Juan Carlos Ojeda-Garces

**Affiliations:** Faculty of Dentistry, Universidad Cooperativa de Colombia, San Juan de Pasto, Nariño, Colombia

## Abstract

This study identifies the most common microorganisms present in type III gold cast posts related to pulpal disease and evaluates the sterilization/disinfection method before cementation in the root canal. Forty-five type III gold cast posts were aseptically collected in sterile sealed plastic bags and taken to the microbiology laboratory to carry out the study: fifteen cast posts had no treatment, fifteen were disinfected (immersion in 70% alcohol during 15 minutes), and fifteen were autoclaved at 121°C for 15 minutes by using saturated steam under 15 psi pressure. By using a two-proportion *z*-test, the difference was statistically significant (*p* > 0.05) and demonstrates that, in spite of the aseptic pattern used in the cast post collection and laboratory procedures, some cast posts arrive contaminated at the consulting office. The disinfection process worked out in a high percentage and demonstrated that the sterilization by autoclaving eliminated completely the pathogenic microbiota without affecting the cast post shape and integrity that could compromise their final fitting.

## 1. Introduction

It is generally accepted that the success of the endodontic treatment correlates positively with the quality of the root canal filling. It is expected that the properly plugged root canals should provide a three-dimension seal against the entry of bacteria [1]. The crown microleakage is an important factor to bear in mind as a triggering factor of the endodontic treatment failure [2]. For years there has been a lot of emphasis on the quality of the final restoration, for which the intraradicular posts are commonly used to achieve the sealing and provide proper crown support [3].

It has been demonstrated that the endodontically treated teeth exposed to the oral cavity are contaminated invariably by the fluids, bacteria, and its by-products. The long-term contamination may lead to the endodontic treatment failure and put the restorative treatment at risk [4–8].

The maintenance of aseptic conditions during the root canal treatment avoids the seepage of bacteria and fluids. However, the sequence of the later root canal preparation procedures, techniques, and instruments used for removal of root canal filling materials [9], the length of the remaining materials, and time of removal of root filling [10], taking of a post space impression, fitting the intraradicular post in the root canal space and post cementation, may allow the unintended contact of the remaining gutta-percha with saliva and bacteria [3, 11–13].

The length of the intraradicular post and the residual filling are also important. The cast post must descend at least two-third of the length of the root in order to provide sufficient retention. If it is not clinically feasible, the post should equal the crown length [14]. Some authors argue that a minimum of 4 mm of filling material must remain in the root canal to avoid bacteria leakage [15, 16]. In other studies, regarding the minimum amount of the remaining filling material [17, 18] it is widely accepted that 5 mm of root canal filling should be left in the apical area in order to avoid disruption of the apical seal in the post space preparation.

It should be considered if it is better to use a permanent high quality restoration immediately after an endodontic treatment or a provisional one that has a greater leakage possibility. The permanence of the provisional cement after the endodontic treatment and before the final cementing, the exposure of the gutta-percha in the filled root canal to mouth liquids, even for brief periods, may lead to treatment repetition before placing the definitive dental restoration. The saliva leakage speed and the microorganisms vary among patients, even from one tooth to another [19]; if a large amount of oral cavity irritants gained access to the periodontal ligament or to the periapical tissue then it may cause inflammation and lead to treatment failure.

At present, more attention is given to the root canal management after the endodontic treatment where it is necessary to have a strict pursuit of the aseptic chain; otherwise microorganisms and their by-products would penetrate into the root canal space and reach the apical area and even the alveolar bone. It is important to understand that, during and after the restoration, the endodontically treated tooth may get contaminated [20].

The aim of this study is to identify the most common microorganisms existent in gold type III cast posts associated with pulpal disease and evaluate the sterilization and disinfection methods before cementation in the root canal in order to establish a protocol that provides with the adequate cast post previous treatment without affecting its shape and function and so avoid the bacterial migration that could affect the prognosis of the endodontically treated tooth.

## 2. Methodology

### 2.1. Sample Preparation

An ex vivo experimental study with 45 lower uniradicular premolars with similar dimensions was carried out. Mesiodistal and linguobuccal X-ray pictures were taken for each tooth to determine similar internal anatomy. Afterwards 15 teeth were distributed randomly in each group. The crowns were removed leaving 2 mm of ferrule effect with hyperflex diamond discs (Ø 18 mm 0.3 mm width). For the endodontic treatment the step-back preparation technique was carried out using the first 25 mm FlexoFile files (Dentsply/Maillefer Instruments SA, Ballaigues, Switzerland) and irrigated with 5.25% sodium hypochlorite and then obturated with gutta-percha cones (Dentsply) and Topseal cement (Dentsply) using the lateral condensation filling technique. After 24 hours the removal of obturation material is performed with Gates-Glidden rotary files (Dentsply/Maillefer Instruments SA, Ballaigues, Switzerland), leaving 4 mm of apical seal. Under aseptic conditions a post pattern was produced in Resin 74 (stodent-INT®) and then was fabricated in gold III (Argenco 18, dental alloy manufacturers).

The cast posts were collected aseptically in sterile sealed plastic bags and taken to the Universidad Cooperativa de Colombia Microbiology Laboratory. They were kept at room temperature (25°C) until the following microbiological analysis was done: (1) microbial quantification in 15 cast posts with no previous disinfection, (2) microbial quantification of 15 cast posts previously disinfected with the conventional method (alcohol 70% during 15 minutes), and (3) microbial quantification of 15 cast posts autoclaved at 121°C (249°F) × 15 lbs of pressure for 15 minutes.

### 2.2. Microbial Isolation

**Figure 1:**
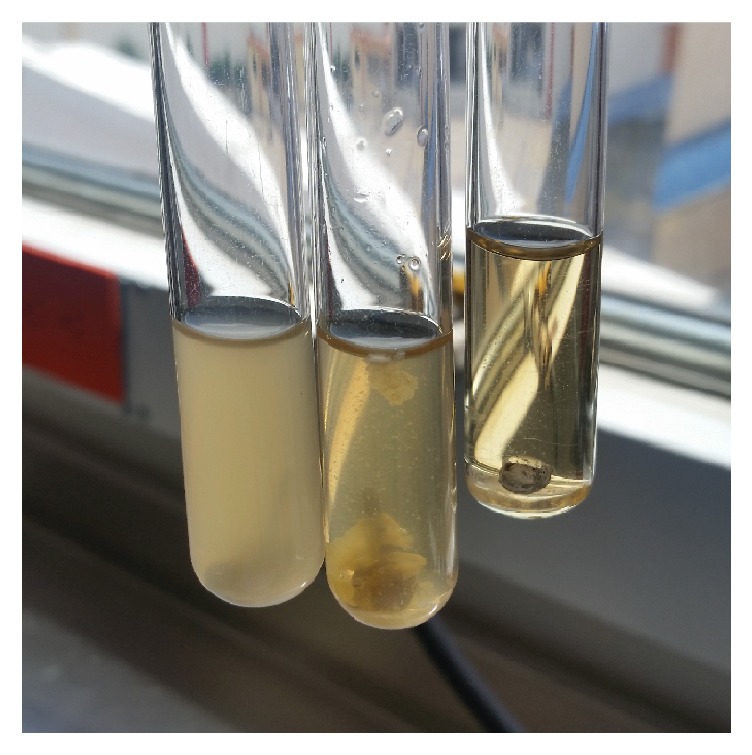
Growth in trypticase soy broth (TSB).

In order to isolate the microorganisms, a general purpose medium was used for growth promotion and subsequently on differential media for the microorganisms' classification. To evaluate the microbiota the Ensinas [21] methodology was followed: the cast posts were immersed individually in 3 ml of trypticase soy broth (TSB, Difco; Becton, Dickinson and Company) and kept at 36.5°C for 7 days (Network line by binder model RI 53). After the first 24 hours a description of turbidity was performed in order to detect the contaminants' presence, by means of turbidity, surface grow, or deposit of material at the bottom of the test tubes (Figure 1).

Growth obtained in this medium was subcultured onto appropriate solid media to obtain pure cultures which afterwards were identified with methods appropriate for the isolates. The positive growth samples were inoculated onto solid media BD Columbia Agar with 5% Sheep Blood and on additional selective and nonselective media.

**Figure 2:**
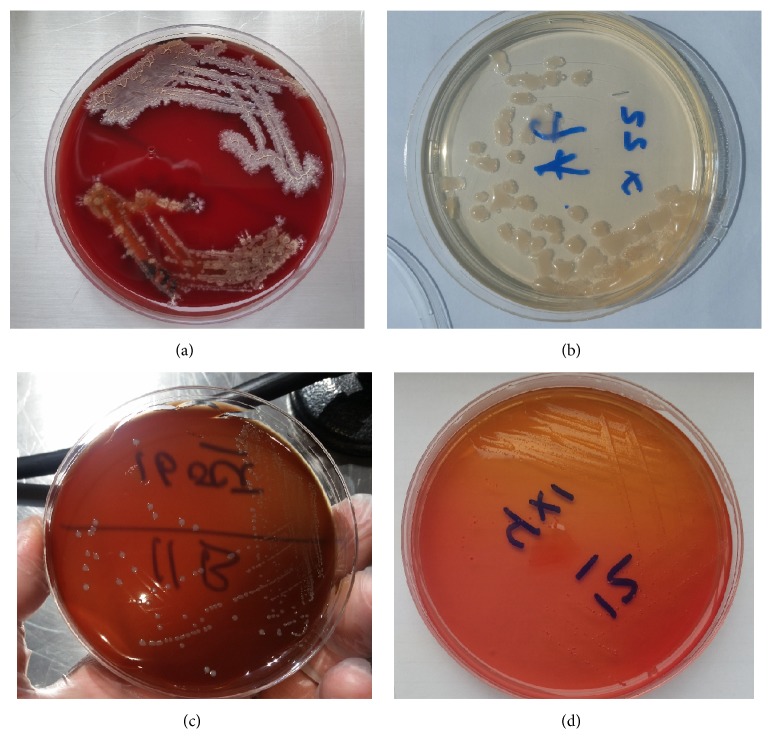
Growth in differential media. (a) Bacteria growing on a blood agar plate. (b) Bacteria growing on Sabouraud agar plate. (c) Bacteria growing on EMB agar. (d) Bacteria growing on Macconkey agar.

From the initial cultures 0,1 ml was transferred into 0,9 ml of peptone broth that corresponds to the 10^2^ dilution; this procedure was repeated until 10^5^ dilution. From the last 3 dilutions 0.1 ml were inoculated in different media: EMB agar, Sabouraud agar, Blood agar, Macconkey agar (Difco; Becton, Dickinson and Company) and incubated at 36.5°C for 5 days (Figures 2(a), 2(b), 2(c), and 2(d)).

### 2.3. Qualitative Description

An examination of cultures of bacteria growing on agar in Petri dishes and colony morphology description was done to identify them. Characteristics such as form, color, size, surface, and texture in bacterial and fungal cultures were outlined for the macroscopic description. The fungal microscopic description was performed by using Lactophenol blue solution (Bioquigen Advanced Chemical).

### 2.4. Microscopic Description Procedure

*Gram Stain*. Slide smears were preparation, fixed, and Gram-stained. The slides were examined to describe shape, color, grouping, and relative size.

### 2.5. Quantification of Microorganisms (Standard Plate Count)

#### 2.5.1. Microdilution and Inoculation

**Figure 3:**
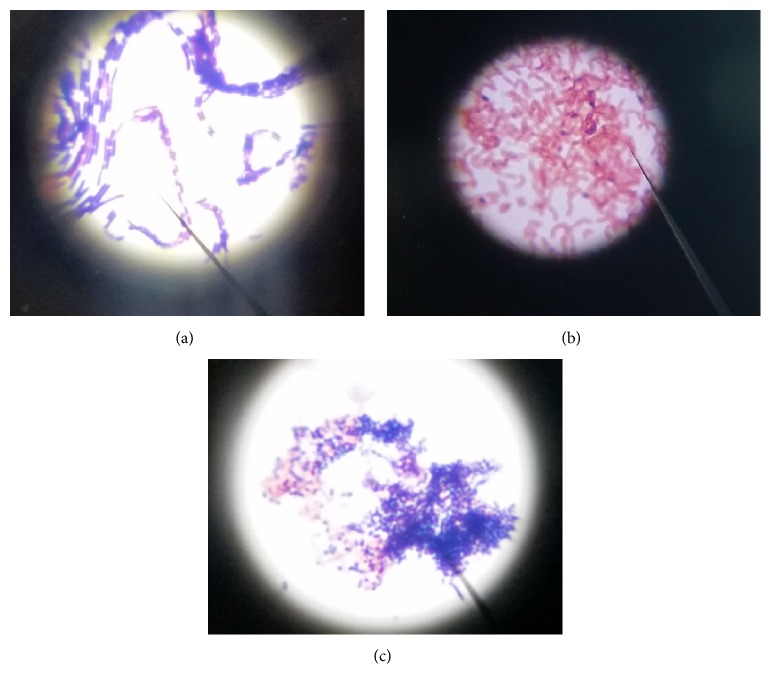
Microscopy images. (a) Gram-positive rods 100x oil. (b) Gram-negative bacilli 100x oil. (c) Gram-positive cocci and Gram-negative bacilli 100x oil.

A quantification of viable cells was done with the serial microdilution technique and the samples spread on top solidified agar. From the chosen colonies, serial decimal dilutions from 10^5^ up to 10^9^ were inoculated on nutritive agar and the viable cells were quantified; the inoculation was made by spreading 0,1 ml of the last three dilutions. The plates were incubated at 36.5°C during 24 to 48 hours. The count of the colonies was done selecting the plates with 30–300 colonies. For the duplicate plates (with same amount plated) the counts were averaged (Figures 3(a), 3(b), and 3(c)).

#### 2.5.2. Primary and Secondary Tests

Gram stain, morphology of bacteria and colonies, catalase test, oxidase test, and biochemical tests.

### 2.6. Statistical Analysis

To statistically analyze the data, a comparison of proportions test was used. The Chi-square test was applied and a 0.018 *p* value was obtained. This *p* value was lower than 0.05. The relationship between the variables treatment and growth was recorded in a contingency table.

## 3. Results

A total of 45 samples were cultured and analyzed. The microorganisms identified were mostly* Staphylococcus* sp.,* Candida* sp., and* Escherichia coli*. Subsequently, the effect of the disinfection and sterilization method on the microbiota of the cast posts was evaluated.

A contingency table was obtained to record the relationship between the variables treatment and growth (Table 1).

The Chi-square test was applied and a 0.018 *p* value was obtained. This *p* value is lower than 0.05 with which the null hypothesis is rejected and concluded that there are differences in the efficiency of the different treatments used for bacterial growth control (Table 2).

Also one concludes that dependence exists between bacterial growth and the type of treatment used to clean. A contingency coefficient of 0,39 indicates that the association is not so strong but there is significance (Table 3).

## 4. Discussion

Many studies have confirmed the importance of the coronal leakage as a possible cause of an endodontic treatment failure since it compromises the seal of the root canal system; therefore, they think that the crown restoration substitutes the missing structure; it protects the tooth remainder from break and avoids the recontamination as the first barrier for the protection of periapical tissues [6, 7, 22, 23].

It has been demonstrated that the oral fluids, bacteria, and its by-products can permeate across the marginal breaches of defective restorations and penetrate into the interface between the filling material and the walls of the root canal, reaching the periapical area in a relatively short time [5–7, 22, 23].

This situation worsens in teeth with extensive crown loss that implies: the fitting of root canal posts, due to the sequence of procedures for the post space preparation, post impression, adjustment of the post in the root canal, and the fitting of temporary restorations, what may allow the accidental contact of the radicular filling with saliva and bacteria [11, 13].

The teeth indicated for posts often result in the alteration of the apical seal during the post space preparation, independently of the method chosen for the elimination of the gutta-percha [24, 25]. So, the search for the ideal root canal obturation material for the teeth indicated for posts and cores is still under discussion.

The polymicrobial origin of the oral infection allows bacterial symbiotic and synergistic phenomena. Until the beginning of the pulpitis, the implied bacteria will be principally aerobic; nevertheless its proliferation will cause anaerobic conditions and neurovascular necrosis of the pulp which in turn favor the conditions for the growth of facultative anaerobic bacteria and, later on, strict anaerobes responsible for the primary infectious processes [26].

*Enterococcus faecalis* is the more frequent pathogen found in asymptomatic endodontic infections [27, 28] due to its aptitude to invade the dentinal tubules, to compete with other microorganisms and to resist nutritional deprivation. It is a facultative anaerobic Gram-positive cocci [29, 30].

*Candida albicans* is an opportunistic fungal pathogen of the oral cavity native biota, but it associates with endodontic infections more frequently than not [31]. It has been demonstrated that it is also present in failed endodontic treatments [32, 33].

In this study, the microorganisms found on the surface of the posts were facultative anaerobic Gram-negative rods (*Escherichia coli*), Gram-positive cocci (*Staphylococcus* sp.), and fungi (*Candida* sp.), associated principally with the cast post handling which leads to a crossed contamination. If is not eliminated before its definitive insertion, they might become responsible for the different types of periapical pathology or refractory injuries to the conventional endodontic treatment.

Several investigations are consistent in that the bacterial filtration is produced independently of the root canal obturation remnant [4–8, 12]. Therefore, an important role of the endodontic obturation is the formation of a physical barrier to prevent bacteria from coming into the apical region and periapical tissues [30].

Also, the immediate and late post space preparation produced similar results in terms of the seal of the root canal and bacterial leakage, independently of the filling and the preparation method used [34, 35].

Nevertheless, in any teeth the residual obturation is not able to prevent the bacterial filtration, probably due to the presence of hollows in the interface between the filling material and the walls of the root canal. These interfacial defects can be due to variables such as the internal anatomy of the dentin tubule system, biomechanical preparation, properties of the root canal irrigants, physical-chemical properties of the endodontic materials, and root canal obturation skills [30].

Torabinejad et al. [7] demonstrated that* Proteus vulgaris* was able to penetrate across the obturated root canal in an average time of 49 days and that* Staphylococcus epidermidis* reaches the root apex within 24 days. Gish et al. [36] reported that* Streptococcus anginosus* penetration happened within 71 days, while Khayat et al. [5] found the bacterial microleakage between 4 and 48 days. Barrieshi et al. [37] checked the bacterial filtration of a mixed anaerobic community of microorganisms in obturated root canals after post space preparation and found that the time of bacterial penetration changed from 48 to 84 days.

In spite of the wide range of root canal obturating materials and current techniques, the results of different studies [8, 14] have demonstrated systematically that none of them is able to prevent completely the coronal or apical filtration, which leads to the concept that a hermetic seal of the root canal is hardly attainable in endodontics.

Other studies to evaluate and analyze the effect of differing irrigation intraroot canal protocols for the post space [38, 39] have been carried out for the post space and its further effect on the adhesion and retention of the posts [40, 41]. For that, the use of chemical agents such as NaOCl, H_2_O_2_, EDTA, chlorhexidine gluconate, citric acid (10%, 20%, and 50%), orthophosphoric acid, and a mixture of them has been proposed, in order to get rid of the smear layer [42], but the scientific literature on the methods to remove the microorganisms from the cast posts before cementation is scarce.

## 5. Conclusions

The casts posts get contaminated during their handling in the dental laboratory; therefore their disinfection, before being definitely cemented in the root canal, is an indispensable requisite to avoid the migration of pathogenic microorganisms inside them.

The sterilization process by autoclaving eliminated completely the pathogenic microbiota without affecting the cast post shape and integrity that could compromise their final fitting.

Advisable measurements include the use of cast posts and adhesive systems to reduce the number of clinical meetings and to avoid the exposition of the obturation to the oral cavity.

## Figures and Tables

**Table 1:** Contingency table treatment *∗* growth.

Count	Growth		Total
	No	Yes	
Treatment			
No treatment	10	5	15
Disinfection	14	1	15
Sterilization	15	0	15

Total	39	6	45

**Table 2:** Chi-square test.

	Value	df	Asymp. sig. (2-sided)
Pearson Chi-square	8,077	2	,018
*N* of valid cases	45		

**Table 3:** Symmetric measures.

	Value	Approx. sig.
Phi coefficient	0,424	0,018
Cramer's V	0,424	0,018
Contingency coefficient	0,390	0,018
*N* of valid cases	45	
